# Electromechanical Performance and Figure of Merit Optimization for Flexible Lead-Free PDMS–*BaTiO*_3_ Piezocomposites

**DOI:** 10.1021/acsomega.4c03856

**Published:** 2024-06-20

**Authors:** Mohamed Dhia Ayadi, Dorra Gassara Hammami, Slim Naifar, Ayda Bouhamed, Chedly Bradai, Olfa Kanoun

**Affiliations:** † Laboratory of Measurement and Sensor Technology (MST), Chemnitz University of Technology, Chemnitz 09126, Germany; ‡ Laboratory of Electro-Mechanic Systems (LASEM), National School of Engineers of Sfax, University of Sfax, Sfax 3038, Tunisia; ¶ Higher Institute of Applied Sciences and Technology of Gabes, University of Gabes, Gabes 6029, Tunisia; § Faculty of Sciences of Gafsa, University of Gafsa, Gafsa 2112, Tunisia; ∥ Laboratory of Measurement and Sensor Technology (MST), Technische Universität, Chemnitz 09126, Germany

## Abstract

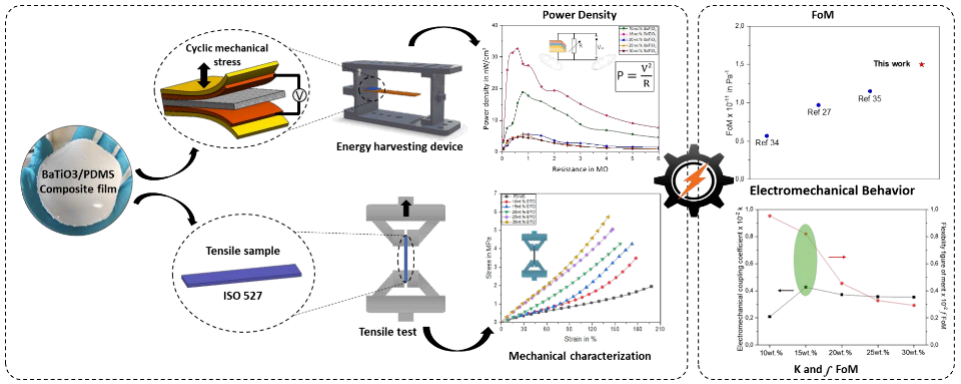

In the modern era of the Internet of Things, the potential role of flexible piezoelectric generators (PEG) reflects the rapid increase in self-powered devices and wearable technologies. In this study, a casting process to elaborate the polydimethylsiloxane (PDMS)/barium titanate (*BaTiO*_3_) composite has been presented. The addition of 15 wt % *BaTiO*_3_ microparticles into the PDMS polymer greatly enhances the piezoelectric coefficient (*d*_31_ = 24 pC N^–1^), leading to an increased output voltage of approximately 4 V under finger tapping force. The proposed flexible microgenerator yielded an excellent piezoelectric figure of merit (*FoM*_31_ = 13.1 × 10^–12^ m^2^ N^–1^), significantly enhancing successfully the energy-harvesting performance (power density of 35 nW/cm^2^). Furthermore, the fabricated lead-free PEG exhibited an excellent flexibility figure of merit (*f*FoM) due to the low young modulus values (Maximum *E* = 3.4 MPa). These results indicate efficient energy conversion and demonstrate a favorable balance between the flexibility and piezoelectric properties of the composite, highlighting its potential for a wide range of applications in self-powered wearable sensors able to collect different human motions in applications such as gesture tracking and finger motion detection.

## Introduction

In the last few decades, piezoelectric materials have found increasing interest in the fabrication of flexible generators, which have shown a wide range of potential applications such as energy harvesting, self-powered biomedical instruments, and wearable electronics. Piezoelectric materials have received great attention due to their ability to effectively convert mechanical energy to electricity. In particular, piezoelectric ceramics have great piezoelectric and dielectric properties, as well as better temperature and chemical stability than other piezoelectric materials.^1^ For that reason, they are frequently employed in generator production. On the other hand, microtechnology and microstructured materials have been used in the design and manufacture of energy harvesters because of the rapid advancement of modern technology.^2^ Piezoelectric generators (PEG) are more portable and adaptable than conventional generators.^3,4^ Due to their great flexibility, stretchability, and ease of manufacture, elastomer polymer-based generators have been extensively explored to fabricate flexible PEG. For instance, the most commonly utilized elastomer polymers are PDMS,^5−7^ PVDF,^8,9^ and PMMA.^10^ As a biocompatible elastomer with the properties of flexibility, transparency, and ease of fabrication, PDMS (polydimethylsiloxane) stands out as a favored option for the fabrication of flexible generators.^11,12^ In addition, due to their low Young’s modulus and improved flexibility when compared to other flexible PEGs manufactured with PVDF, PDMS-based composite films are more suitable for conformal applications like e-skin, and also exhibit better charge response and power output.^13,14^ In order to improve the flexibility and the softness of PEG, enhancing the mechanical and physical properties of the composite materials is a requirement.^15^ Moreover, the improvement of the energy harvesting and power output performance of a PEG is related to piezoelectric particles with a high dielectric constant exhibiting a high voltage response.^16^ For the selection of piezoparticles, a variety of materials have been used to fabricate generators, such as ZnO,^17,18^ BCZT,^19^*BaTiO*_3_ (BTO),^5,20−22^ and KNLN.^23^ However, the focus was principally on *BaTiO*_3_ piezoelectric particles due to their high dielectric constant and great biocompatibility,^5,24^ to improve the output performance of flexible generators with an excellent figure of merit (FoM), high sensitivity, and long durability for self-powered sensors.^25−27^

For example, in ref (28) PDMS-based piezoelectric composites filled with ferroelectric *BaTiO*_3_ (BTO) particles were fabricated at different BTO concentrations (20, 30, 40, and 50 wt %). Sappati et al. have demonstrated that these films are very flexible with a very low Young modulus compared to PVDF. A 50 wt % BTO film achieves a dielectric constant of 4.59 and a Young’s modulus of 3.92 MPa. Besides, Meng et al.^12^ have focused on improving the dielectric and morphological performance of the *BaTiO*_3_-based PDMS matrix with different particle sizes at different concentrations (from 5 to 40 wt %). The dielectric constant and dielectric loss of the BTO/PDMS composite films were investigated. The performance of (triboelectric generator) TEG and (piezoelectric generator) PEG was improved by increasing the number of BTO particles in the composite. It was found that the 40 wt % BTO/PDMS composite achieved a high dielectric constant and low dielectric loss value (0.12). In addition, to ensure that the PEG produces better output performance for energy harvesting applications (flexible and wearable electronics) while being environmentally friendly, Mahanty et al.^29^ have investigated a lead-free ZnO nanoparticles (NPs) reinforced poly(vinylidene fluoride) (PVDF) composite generator, and the PEG exhibits great piezoelectric charge coefficient and figure of merit values (*d*_33_ = 33.6 pC·6*N*^–1^, FoM = 12.7 × 10^–12^ Pa^–1^).

Most of the ongoing research on piezoelectric generator (PEG) devices for energy harvesting has mainly concentrated on their electrical behavior and mechanical characteristics such as power output and flexibility. However, studies on the correlation between the electrical and mechanical properties of PDMS/*BaTiO*_3_, through the figure of merit (FoM) and the electromechanical coupling factor for energy harvesting, are still not well investigated. So, This study aims to investigate and optimize the electromechanical coupling factors and piezoelectric figure of merit of PEG based on varying concentrations of *BaTiO*_3_ particles. *BaTiO*_3_–PDMS composite piezoelectric thin films are fabricated by using a casting process, and their mechanical properties are obtained through tensile tests. The electrical response of the PEG is experimentally determined, and the figure of merit is calculated through theoretical analysis. The paper presents first the casting process and PEG structure preparation. Second, results and discussions on mechanical behaviors and generator performance have been developed utilizing the figure of merit (FoM) investigation. Finally, a conclusion is presented with suggestions for additional research.

## Materials and Experimental Details

In this work, a PDMS/*BaTiO*_3_ composite material was chosen for energy harvesting applications due to PDMS’s polymer elastomeric properties and high flexibility,^30^ as well as *BaTiO*_3_’s excellent dielectric and piezoelectric properties. The casting process is performed because of the simplicity and efficiency of the method, allowing for the development of flexible composite generators. Utilizing a specific testing device based on resonance frequency vibration enabled precise characterization, providing an important addition to improvements in piezoelectric generators.

### Materials

In this study, the used *BaTiO*_3_ was purchased from Sigma Aldrich and is composed of 99.5% trace metals with particle sizes less than 2 μm. Due to their great integration at the polymer matrix,^5^ these microparticles are used to reinforce the composite generators with different concentration values (10, 15, 20, 25, and 30%). PDMS (two-part Dow Corning Sylgard-184 silicone elastomer) was purchased from Mavom GmbH and is used as a matrix for composite films. Moreover, Table 1 shows the main properties of *BaTiO*_3_ piezoparticles and the PDMS polymer that were added to the mixture during the elaboration step. Additionally, tetrahydrofuran (THF) is an aprotic solvent with a dielectric constant of *ε* = 7.43.^31^ It allows the particles to be well dispersed in the polymer matrix of the composite.

**Table 1:** Properties of Materials^31−33^

Properties	Barium titanate (*BaTiO*_3_)	Polydimethylsiloxane (PDMS)	Tetrahydrofuran (THF)
Physical state	Powder	Liquid	Liquid
Color	White	Colorless	Colorless
Melting point	21.8–42 °C	-	-
Formula weight	233.19 g/mol	27 g/mol	72 g/mol
Density	6.08 g/cm^3^	1.11 g/cm^3^	0.8876 g/cm^3^
Size	<2.0 μm	-	-
Young’s modulus	50 to 80 GPa	0.8 to 2 MPa	-
Dielectric constant	100 to 200	2.8 to 3.2	7.43

### Fabrication Process of the Piezoelectric Generator

**Figure 1:**
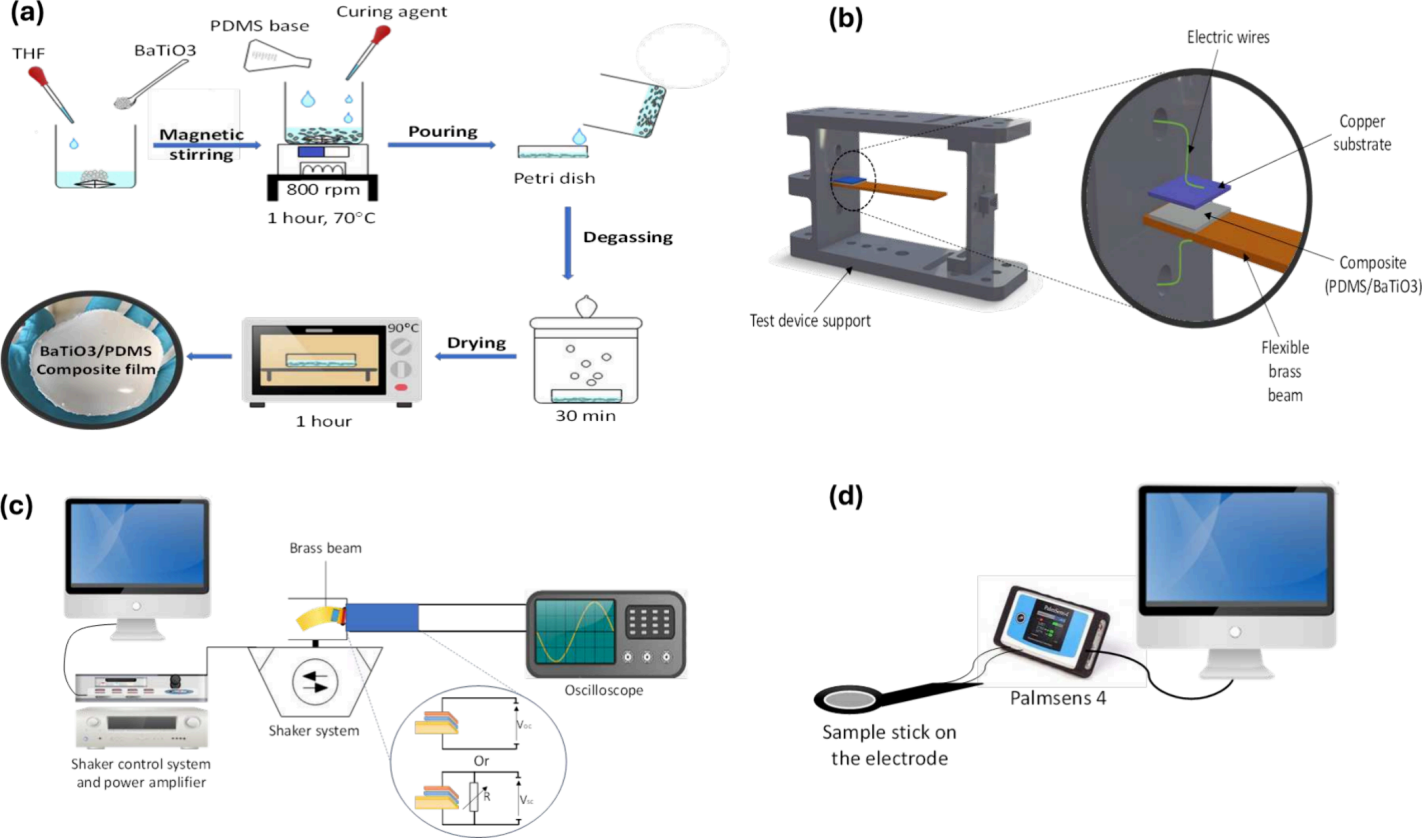
Fabrication process and experimental characterization of PEG: (a) Flowchart of elaboration process of PDMS-BT composite. (b) Preparation of the PEG structure. (c) Energy harvesting experimental setup for open circuit (*V_oc_*) and short circuit (*V_sc_*). (d) Impedance experimental setup.

To fabricate the piezoelectric composite generators, *BaTiO*_3_ microparticles (10, 15, 20, 25, and 30 wt %) were solved in THF, and the solution was kept stirring for 1 h at 70 °C. Then, PDMS was added to the solution, which was kept stirring for 1 h at 70 °C (PDMS base polymer and curing agent were added in a weight ratio of 10:1). In the last 10 min, the curing agent was added. The stirring speed is 800 rpm. In order to remove bubbles, the solution was poured into the Petri dish and kept degassing for 30 min, as shown in Figure 1a. The solution was then placed in a 90 °C oven for 1 h to cure the PDMS and evaporate the THF solvent from the mixture. Finally, the composite film was peeled off from the mold.

To produce the piezoelectric generator (PEG), a sandwich structure is performed that is composed of three layers consisting of two conductive layers (a copper substrate sized 1.5 cm^2^ and a brass beam sized 5.5 × 2 cm) and a composite layer (size 1.5 × 1.5 cm^2^) between them as shown in Figure 1b. However, the bond between the composite layer and the electrodes is based on one hand, and the brass beam–composite layer adhesion was ensured by pouring the composite solution on the brass beam. This can ensure a strong bond between the brass beam and the composite layer, which allows efficient stress transfer. On the other hand, the copper substrate–composite layer adhesion was realized by bonding the copper substrate to the top surface of the composite layer. To achieve this, a tape was used to adhere the copper substrate to the composite layer. This method provides a reliable connection between the copper substrate and the composite layer. Hence, the idea is to ensure similar operating conditions during the experimental measurements conducted for all PEGs.

### Experimental Investigations

In order to investigate the performance of the PEGs, the experimental setup used consisted of an electrodynamic shaker (VebRobotron Type 11077), which is used to provide a harmonic vibration, as shown in Figure 1c. The applied excitation is controlled using a laser USB shaker control system and a linear power amplifier (LDS LPA 100). The acceleration sensor (ADXL 326) is mounted on the moving part of the system. Moreover, the output signal response is illustrated by a digital oscilloscope (LeCroy WaveRunner 6050A).

For the tensile test, an Instron universal testing machine (Electro Puls E10000) is used. Film samples are cut into 35 mm × 5 mm rectangular shapes following the standard ISO 527. Tensile testing was carried out at a speed of 5 mm/min. These tests define the mechanical properties of composite generator films at all concentrations.

The impedance tests are based on the PalmSens4 instrument involved in measuring the dielectric response at each *BaTiO*_3_ concentration in the composite as shown in Figure 1d. Moreover, these tests allowed the determination of the piezoelectric voltage constant (g) by analyzing the material’s behavior at various percentages by weight. This method provided data about the material’s dielectric properties, allowing for an improved characterization of the generator composites.

## Results and Discussion

### Morphology and Flexibility of Composite Generator

**Figure 2:**
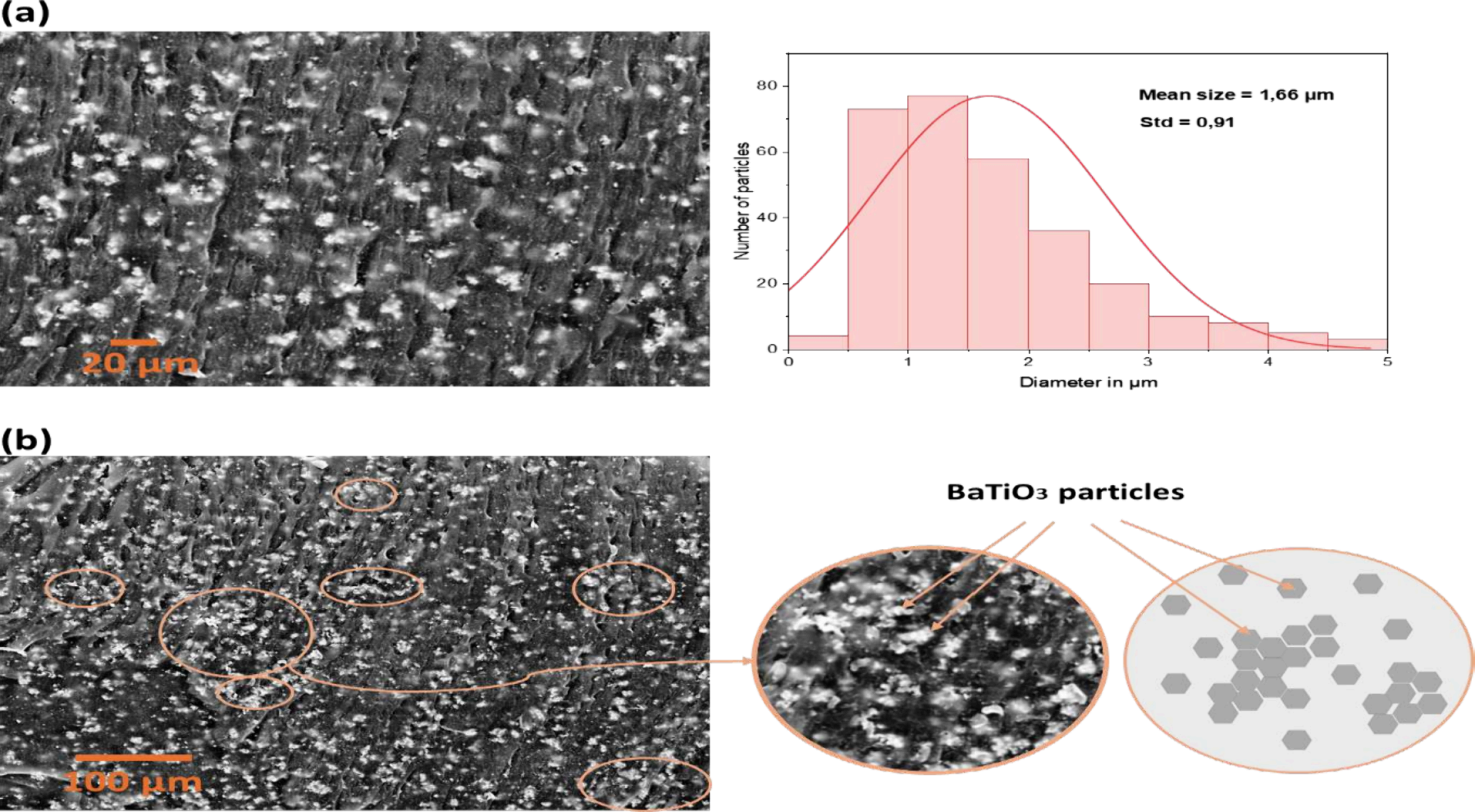
Cross-sectional SEM images of the composites: (a) 15 wt % *BaTiO*_3_ distribution within the PDMS polymer with the average size distribution of the particles and (b) 30 wt % *BaTiO*_3_/PDMS image and illustration of the inhomogeneity of the composite.

The cross-sectional morphological SEM images of the 15 wt % BTO and 30 wt % BTO composites are presented in Figure 2. SEM is performed to capture such images in order to verify the mean size of particles and the uniformity of the particle distribution. However, the cross-sectional SEM image of 15 wt % BTO microcomposite is illustrated in Figure 2a. The image reveals the uniform distribution of BTO particles inside the polymer matrix and it clearly proves the homogeneous microstructure. Additionally, based on SEM image analysis the BTO microparticles have an average size of 1.66 μm. Furthermore, as observed in Figure 2b, many agglomerates formed in the PDMS matrix as a result of the 30 wt % *BaTiO*_3_ concentration. Hence, the composite was sutured with BTO microparticles and is not homogeneous in structure anymore. Therefore, at low concentrations, the microparticles of BTO have more free space, but at high concentrations, microparticles will be forced to bond particle to particle and create agglomeration within the polymer.

**Figure 3:**
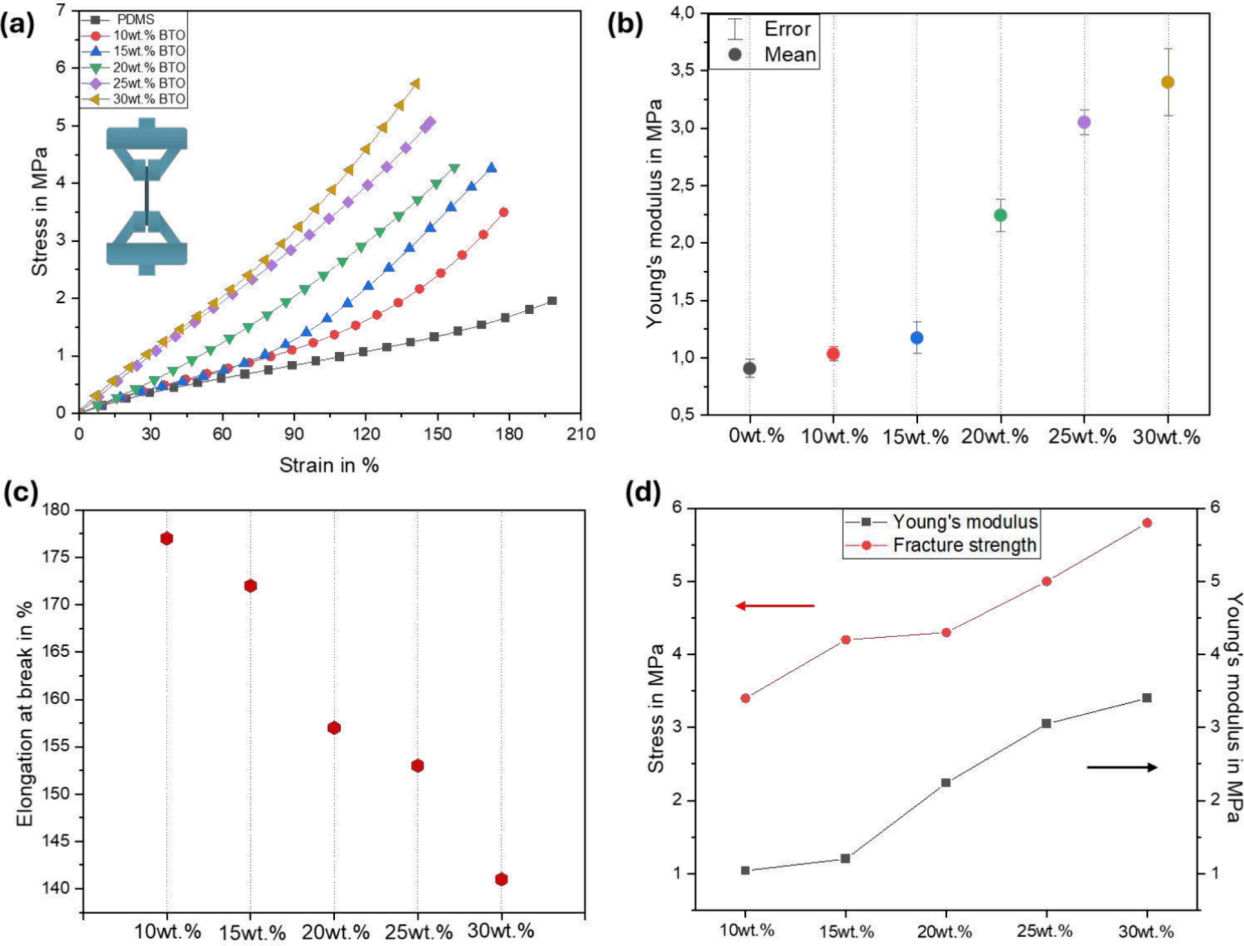
Mechanical properties of PEG composites: (a) Stress–strain behavior as a function of *BaTiO*_3_ concentration. (b) Error bar of Young’s modulus of PDMS–*BaTiO*_3_ composite films. (c) Elongation at break of BTO/PDMS composites as a function of *BaTiO*_3_ concentration. (d) Comparison of Young’s modulus and fracture strength of *BaTiO*_3_ concentrations.

Beside the material morphology study, mechanical behavior based on tensile testing was performed to investigate the flexibility of BTO/PDMS composites at different concentration levels. Therefore, stress–strain graphs were obtained for all composite concentrations (10, 15, 20, 25, and 30 wt %), as shown in Figure 3a. The results show that fracture strength increases of approximately 60% were observed from 3.6 to 5.8 MPa with the 10 wt % of *BaTiO*_3_ in comparison to the 30 wt % in the mixture. The elongation at break of 30 wt % *BaTiO*_3_ has decreased by around 22%, from 177% to 144% compared to 10 wt % mixture as shown in Figure 3c. This fall is due to the material becoming less flexible and more fragile over time. Thus, the results demonstrate that when ceramic content increases, tensile strain decreases, and film fragility increases.

Furthermore, tensile tests have been developed for all *BaTiO*_3_ concentrations in order to determine their young modulus values, as illustrated in the error bar in Figure 3b. By introducing *BaTiO*_3_ particles of varying weight percentages into the PDMS polymer (10, 15, 20, 25, and 30 wt %), Young’s modulus of the composite experienced significant enhancements. Specifically, the addition of 10% increased Young’s modulus by approximately 15% compared to pure PDMS polymer, pushing it from 0.9 to 1.04 MPa. Adding 15 wt % resulted in a 35% increase, elevating the modulus to 1.2 MPa. An important improvement of 150% was observed when 20% was added, raising the modulus to 2.24 MPa. Further increases of 240% and 280% were reached with 25 wt % and 30 wt %, respectively, leading to Young’s modulus of 3.05 and 3.4 MPa.

These findings highlight the direct relationship between the quantity of *BaTiO*_3_ particles and the improved stiffness and Young’s modulus of the composites as shown in Figure 3d. Therefore, these films are extremely flexible (elongation more than 170%, Figure 3c), with a maximum Young’s modulus value of 3.4 MPa. According to these results, Young’s modulus is obviously dependent on the ceramic component in the piezoelectric composite films. It is worth noting that a high Young’s modulus restricts efficient stress transmission, reduces the open circuit voltage caused by mechanical stress, and contributes to the rigidity of PEGs.

### Energy Harvesting Performance at Low Deformation

**Figure 4:**
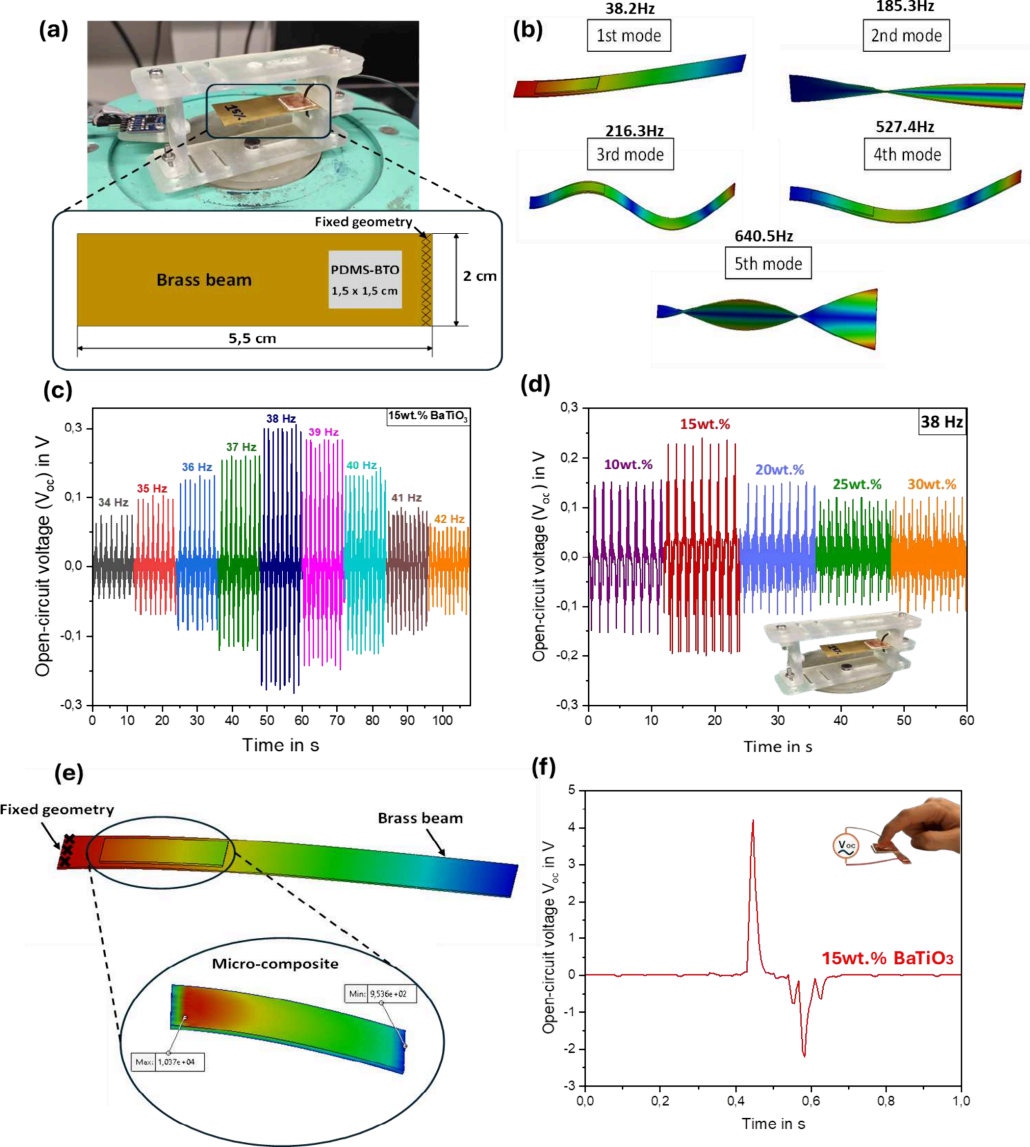
Piezoelectric microgenerators output performances: (a) Detailed illustration of the microgenerator. (b) Resonance frequency simulation for the PEG. (c) Open-circuit output voltage (*V_oc_*) of 15 wt % BTO composite at different frequency levels. (d) Open-circuit voltage values (*V_oc_*) depend on the BT concentration at 38 Hz frequency. (e) 3D model of stress applied to composite. (f) Test under finger tapping of 15 wt % BTO.

To determine the resonance frequency in the first mode of the PEG structure, a frequency study is performed based on resonance frequency simulation on the SolidWorks software. The resonant frequency simulation includes a 3D model of the geometric dimensions of the PDMS–BTO composite microgenerator (1.5 × 1.5 cm) bonded to the brass beam (5.5 × 2 cm) as illustrated in Figure 4a. In addition, the specific material constants including Young’s modulus, Poisson’s ratio, and mass density are considered in each material property. Furthermore, the 3D model should take into account the boundary conditions to replicate the real experimental scenarios by imposing a fixed geometry at the end of the beam, as it is fixed in reality in the testing device. Therefore, the simulation results reflect the influence of material properties on the resonant frequency of the microgenerator, so it must converge to an average Young’s modulus value of the five BTO concentrations (average *E* = 2.2 MPa). Figure 4b shows values of different resonant frequencies at five proper modes, so it can be seen that the value of the resonance frequency at the first mode is 38.2 Hz, and this value is a requirement for this study.

The resonance frequency of the harvesting test device can be observed as shown in Figure 4c, which corresponds to the highest voltage output produced by the sample containing 15 wt % BTO. The value of this frequency (38 Hz) can be taken into consideration to evaluate the performance of all the concentrations of *BaTiO*_3_ in the composite generators (10, 15, 20, 25, and 30 wt %) using a test device support (Figure 4a), which is fixed on a shaker system.

Figure 4d indicates the open-circuit voltage obtained by different PEGs under mechanical deformation of the brass beam during the harmonic vibration test at the resonance frequency (38 Hz) and with a peak of acceleration at 4g (4g = 39.2266 m/s^2^). In fact, during the vibration test, the composite PEG bonded on the brass beam will be deformed due to the cyclic compression–extension stress applied according to the X axis (σ_*a*_). This applied stress can be estimated based on a 3D model simulation as shown in Figure 4e. In the 3D model, the obtained resonance frequency (38 Hz) was applied to the composite bonded in the brass beam to observe the maximum stress transferred to the PDMS-BTO composite. Through this process, the mechanical stress transmitted to the composite was determined (approximately σ_*a*_ = 10 kPa). However, the transmitted stress leads to orientation of the electric dipoles. As a result, the piezoelectric response and electrical output will be exhibited. When the amplitude of the deformation is low enough to reach zero and the mechanical deformation is removed from the composite, the latter is released, including the return of the piezoelectric charge to its original state, which reverses the output voltage and current. Consequently, an alternative output voltage is obtained. In this case, the open-circuit voltage (voltage) of different concentrations is obtained. In fact, Equation 1 can express the piezoelectric voltage output:
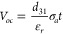
1where *d*_31_, *ε*_*r*_, *t*, and σ_*a*_ are the piezoelectric charge coefficient, relative dielectric constant, film thickness (100 μm), and mechanical stress, respectively.

According to these results, the generators containing 15 wt % *BaTiO*_3_ exhibit a high output voltage of around 0.25 V under low stress (σ_*a*_= 10 kPa), which is around two times higher than those of the sample prepared with 10 wt %. The increased voltage output suggests that the composite’s 15 wt % *BaTiO*_3_ concentration can provide more electrical energy than the other concentrations. On the other hand, the lowest output voltage is produced when *BaTiO*_3_ is present in a 30 wt % concentration due to the agglomeration of the piezoelectric particles and the oversaturation of dipoles inside the composite. This shows that the performance of the generator is dependent on the concentration of *BaTiO*_3_ in the composite. As a consequence, the high electrical performance of the PEG is due to the good dispersion of the piezoelectric microparticles as well as the optimal piezoelectric dipoles in the polymer at this concentration, which are the two key factors for obtaining a higher electrical output response in PEG (15 wt % of *BaTiO*_3_).

In order to validate the piezoelectric potential of the PEG for practical applications, the NG was tested under finger tapping, as illustrated in Figure 4f. The 15 wt % *BaTiO*_3_ NG proved the ability to harvest biomechanical energy up to 4 V. As a result, enhanced voltage response leads to increased generated power, highlighting the essential role of piezoelectric microparticle optimization and figure of merit analysis.

### Power Output Performance and Figure-of-Merit (FoM) Enhancement

**Figure 5:**
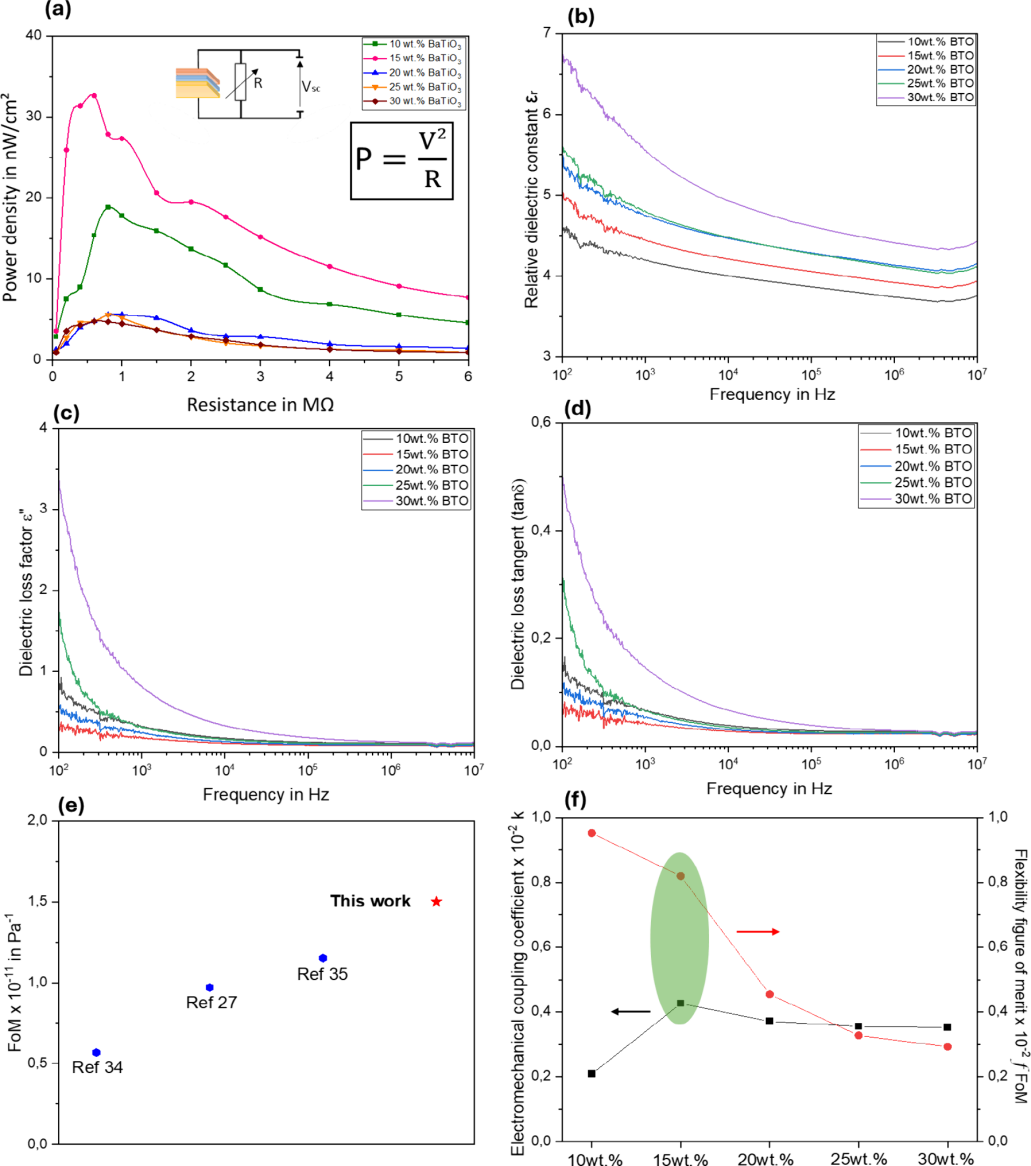
Power output and figure-of-merit (FoM) characteristics of PEG: (a) Power density depending on *BaTiO*_3_ concentration. (b) Relative dielectric constant of PEG. (c) Dielectric loss factor of each *BaTiO*_3_ concentration level. (d) Dielectric loss tangent as a function of *BaTiO*_3_ weight percent. (e) Comparison of piezoelectric figure-of-merit with recent works. (f) Flexibility figure-of-merit and electromechanical coupling factor behavior.

To study the quality of the generator and investigate the power produced by the PEGs at the short circuit, an experimental test was done under a sweep of resistances from 50 kΩ to 6 MΩ in order to define the output power of the PEGs under compression-extension stress applied according to the X axis (σ_*a*_), and the results are shown in Figure 5a. In fact, these values represent the electrical power that the PEG can produce when related to different load resistances.

According to these results, the highest power density can be produced by the 15 wt % of *BaTiO*_3_ generator composite of W = 35 nW/cm^2^ at a load resistance around of 600 kΩ. It demonstrates that the optimal resistance to load for increasing power transfer from the generator to an external circuit is 600 kΩ. However, the power output decreases with increasing load resistances (800 kΩ and 900 kΩ), suggesting a decrease in the electrical power production from the generator. This can be caused by a decrease in the current that flows through the load resistance, which results in a loss of power. Consequently, the *BaTiO*_3_ concentration of 15 wt % is found to provide the maximum power output in the PDMS/*BaTiO*_3_ generator composite, showing that the output voltage and internal resistance are well balanced. The highest electrical power output is generated because of this concentration, which produces a perfect combination of internal resistance and piezoelectric properties. Overall, it has been found that the 15 wt % *BaTiO*_3_ concentration in the PDMS/*BaTiO*_3_ generator composite is the concentration that is optimal for achieving the highest power output due to an ideal combination of internal resistance and piezoelectric characteristics due to the excellent value of piezoelectric charge constant (d) and high figure of merit (FoM). Therefore, the correlation between the mechanical behavior (Exp: Young’s modulus (E)) and the electrical output response for PEGs is based on the piezoelectric figure of merit (FoM).^34^

Further, to analyze the performance of the NG, the piezoelectric figure of merit (FoM = *g*_31_·*d*_31_) was estimated for the composite PEG. The estimated piezoelectric voltage coefficient was found approximately as *g*_31_= 0.55 V m N^–1^ according to Equation 2.
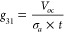
2

Moreover, PEG is evaluated by measuring the piezoelectric charge constant corresponding to the X-axis (*d*_31_) after obtaining the relative dielectric response (*ε*_*r*_) of each concentration as shown in Figure 5b.

In addition, Figure 5c shows the dielectric loss factor, which measures the energy dissipated within a material when it is exposed to an electric field. The dielectric loss tangent is also shown in Figure 5d to understand the dielectric efficiency of the composites. Mathematically, it is expressed as follow:
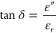
3

At low *BaTiO*_3_ concentrations, the microparticles are well dispersed in the PDMS matrix, which improves the overall dielectric properties. This is due to minimal interparticle interactions and maintained polymer flexibility. As the concentration of *BaTiO*_3_ increases up to 15%, the dielectric loss and tangent decrease, suggesting an optimal filler content where the composite exhibits the optimum balance of low energy dissipation and improved dielectric properties.^35^ At higher concentrations above 15%, agglomeration of *BaTiO*_3_ particles can behave as defect regions leading to localized electric fields and increased dielectric losses.^36^ This coefficients (*ε*_*r*_, *ε*″, and tan δ) were obtained using experimental impedance tests. Thus, 15 wt % of *BaTiO*_3_–PDMS generator exhibited a high piezoelectric charge constant around *d*_31_ = 24 pC N^–1^ from Equation 4, which makes the enhanced PEG provide excellent *FoM*_31_ and that leads to performed energy harvester microgenerator.
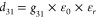
4
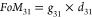
5

Additionally, based on Equation 5, the composite-based PEG exhibited a great piezoelectric figure of merit (*FoM*_31_ = 13.1 10^–12^ m^2^ N^–1^) compared to the P(VDF-TrFE)/MOF composite^37^ and pure P(VDF-TrFE) Mg nanofibers-based PEGs^29^ as well as other various recently reported PEGs^29,38,39^ as shown in Figure 5e. Moreover, Equation 6 can be used to calculate the electromechanical coupling factor *k*, which combines the piezoelectric and mechanical characteristics, in order to determine the ideal quantity of *BaTiO*_3_ in the composite materials.
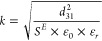
6where *S*^*E*^ presents the elastic compliance (inverse of the young modulus 1/*E*) in m^2^/N. Therefore, the coupling factor (*k*) is based on the mechanical properties and the dielectric performance. Furthermore, materials with a higher flexible figure of merit (σ_*y*_/*E*) are more flexible. Higher *f*FoM can be achieved in an elastic material like elastomer with a low young modulus (*E*).^40^ A material with great strength (high yield stress, σ_*y*_) can be flexible if not too rigid (high *E*). Equation 7 describes precisely the relationship between soft-stiff and weak-strong.
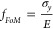
7According to the coupling factor (*k*) values shown in Figure 5f, the composite generator with a 15% concentration of *BaTiO*_3_ has the maximum coupling factor, which shows that it can effectively transform mechanical energy into electrical energy. Moreover, lower coupling factors for the other concentrations indicate that they are less effective at transforming mechanical energy into electrical energy. Additionally, the flexible figure of merit (*f*FoM) describes especially the flexibility of the materials, Hence, increased particles concentration in the PDMS-based composite is directly related to reduced *f*FoM values; lower values provide stiffer material and are unsuitable for this purpose.

A higher piezoelectric charge constant suggests an increased output voltage for a specific mechanical deformation, but a higher Young’s modulus indicates a stiffer and more deformation-resistant material.

Overall, the results indicate that 15 wt % of *BaTiO*_3_ is the ideal level of concentration among the 5 elaborated percentages for a PDMS/*BaTiO*_3_ generator composite to produce the greatest values of power because it highlights a good balance between the piezoelectric and flexibility figure of merit (*FoM*_31_, *f*FoM) and the electromechanical coupling factor (*k*) as shown in Figure 5d. Therefore, the highest power output and coupling factor are produced by this concentration, which demonstrate an effective conversion of mechanical energy into electrical energy.

In summary, optimizing the concentration of *BaTiO*_3_ in the composite is essential for achieving a proper balance between mechanical and piezoelectric properties, which will enhance the performance of composite generators based on the PDMS/*BaTiO*_3_ material combination. By doing this, it is possible to attain a higher figure of merit and, consequently, higher electrical power output, which can encourage the development of energy harvesting systems that are more productive.

## Conclusion

In this paper, the electromechanical coupling factor (*k*) and the figure of merit (FoM) were successfully verified to evaluate and enhance the PEG’s properties. This work highlights the elaboration of lead-free PEGs using a flexible polymer (PDMS) and barium titanate (*BaTiO*_3_) microparticles based on the analysis of various *BaTiO*_3_ concentrations. The results demonstrated that the maximum FoM (*FoM*_31_ = 13.1 10^–12^ m^2^ N^–1^) and power output approximately of *W* = 35 nW are achieved at a concentration of 15 wt % *BaTiO*_3_. In considering this, it can be concluded that the PEG efficiently converts mechanical energy into electrical energy while maintaining an accurate balance between its flexibility (*E* = 1.2 MPa) and piezoelectric properties (*d*_31_ = 24 pC N^–1^). Overall, the research offers useful suggestions for improving energy harvesting technology by validating the importance of the figure of merit on the PDMS–*BaTiO*_3_ biocompatible composite. Enhancing the *BaTiO*_3_ concentration in PEGs can result in more effective and productive devices for a variety of applications. In conclusion, this study successfully contributes to our knowledge of PEGs and their potential for converting mechanical energy into electrical energy and opens the door for future developments by improving the piezoelectric charge response by using polarized PEG composites.
